# RNA-Seq Transcriptomic Responses of Full-Thickness Dermal Excision Wounds to *Pseudomonas aeruginosa* Acute and Biofilm Infection

**DOI:** 10.1371/journal.pone.0165312

**Published:** 2016-10-28

**Authors:** S. L. Rajasekhar Karna, Peter D’Arpa, Tsute Chen, Li-Wu Qian, Andrea B. Fourcaudot, Kazuyoshi Yamane, Ping Chen, Johnathan J. Abercrombie, Tao You, Kai P. Leung

**Affiliations:** 1 Dental and Craniofacial Trauma Research and Tissue Regeneration Directorate, US Army Institute of Surgical Research, JBSA Fort Sam Houston, Texas, United States of America; 2 US Army Center for Environmental Health Research, Fort Detrick, Maryland, United States of America; 3 The Geneva Foundation, Tacoma, Washington, United States of America; 4 The Forsyth Institute, Cambridge, Massachusetts, United States of America; Meharry Medical College, UNITED STATES

## Abstract

*Pseudomonas aeruginosa* infections of wounds in clinical settings are major complications whose outcomes are influenced by host responses that are not completely understood. Herein we evaluated transcriptomic changes of wounds as they counter *P*. *aeruginosa* infection—first active infection, and then chronic biofilm infection. We used the dermal full-thickness, rabbit ear excisional wound model. We studied the wound response: towards acute infection at 2, 6, and 24 hrs after inoculating 10^6^ bacteria into day-3 wounds; and, towards more chronic biofilm infection of wounds similarly infected for 24 hrs but then treated with topical antibiotic to coerce biofilm growth and evaluated at day 5 and 9 post-infection. The wounds were analyzed for bacterial counts, expression of *P*. *aeruginosa* virulence and biofilm-synthesis genes, biofilm morphology, infiltrating immune cells, re-epithelialization, and genome-wide gene expression (RNA-Seq transcriptome). This analysis revealed that 2 hrs after bacterial inoculation into day-3 wounds, the down-regulated genes (infected vs. non-infected) of the wound edge were nearly all non-coding RNAs (ncRNAs), comprised of snoRNA, miRNA, and RNU6 pseudogenes, and their down-regulation preceded a general down-regulation of skin-enriched coding gene expression. As the active infection intensified, ncRNAs remained overrepresented among down-regulated genes; however, at 6 and 24 hrs they changed to a different set, which overlapped between these times, and excluded RNU6 pseudogenes but included snRNA components of the major and minor spliceosomes. Additionally, the raw counts of multiple types of differentially-expressed ncRNAs increased on post-wounding day 3 in control wounds, but infection suppressed this increase. After 5 and 9 days, these ncRNA counts in control wounds decreased, whereas they increased in the infected, healing-impaired wounds. These data suggest a sequential and coordinated change in the levels of transcripts of multiple major classes of ncRNAs in wound cells transitioning from inflammation to the proliferation phase of healing.

## Introduction

*Pseudomonas aeruginosa* is an opportunistic and major nosocomial pathogen that infects wounds [1], including chronic non-healing and combat wounds. These infections can delay wound closure, cause hypertrophic scarring, and become life-threatening [2, 3]. Currently, the interaction between bacterial pathogens and the skin wounds they infect is incompletely understood**.**

As *P*. *aeruginosa* adapts to thrive in the wound, immune cells infiltrate the wound, and resident cutaneous cells in the crossfire try to adapt to the resulting stress, and are either killed or survive to participate in healing. We hypothesized that the transcriptome of the combined cells of the wound tissue countering *P*. *aeruginosa* infection—first active infection and then late-stage biofilm-predominant infection—can provide insight into mechanisms occurring during these phases of infection.

To evaluate this hypothesis, we used a dermal full-thickness, rabbit ear excisional wound model for its easily quantifiable healing end points and its clinical relevance as recognized by the U.S. Food and Drug Administration [4]. Using this model, we and others previously demonstrated that bacterial infections that transitioned from active planktonic to biofilm growth caused delays in granulation tissue in-growth and re-epithelialization [3, 5]. Furthermore, we previously compared wounds infected with *P*. *aeruginosa* with wounds infected with *Klebsiella pneumoniae*, a less virulent bacterium, for transcriptomic responses of the wound and wound-proximal skin using microarrays. Compared to *Klebsiella*-infected wounds, *Pseudomonas*-infected wounds showed gene expression signatures of more intense inflammation, with the cutaneous cells exhibiting signatures of a more severe integrated stress response [3, 6].

Herein we used RNA sequencing (RNA-Seq) to generate transcriptomes because it sensitively and accurately quantifies transcripts, can delineate transcript boundaries and identify novel species of small or non-coding RNAs [7–9]. The wounds whose RNA-Seq transcriptomes were analyzed were also characterized regarding bacterial counts, biofilm morphology, and wound infiltration of polymorphonuclear leukocytes (PMNs), monocytes and macrophages. We found suppressed expression of ncRNAs as the earliest response of the resident cutaneous cells to active infection which preceded a general down-regulation of skin-enriched gene expression. Subsequently, when the chronic biofilm-infected wounds were half closed while non-infected control wounds were almost fully closed, the biofilm-infected wounds showed higher expression of ncRNAs.

## Results

### Study design

**Fig 1:**
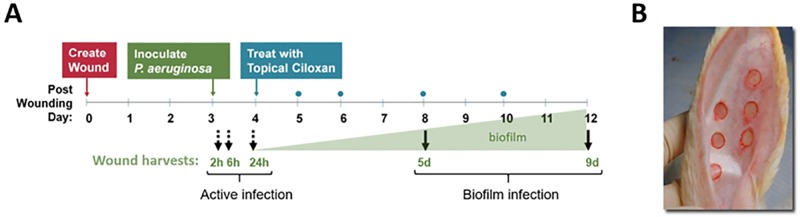
Study design. **(A)** Wounds were infected with *P*. *aeruginosa* (10^6^ CFU/wound) or were sham-infected (vehicle control, phosphate buffered saline, PBS) on post-wounding day 3, and were then harvested 2, 6, or 24 hrs later (dotted arrows, Active infection). Other wounds that were similarly infected or sham-infected for 24 hrs subsequently received topical ciprofloxacin ointment (Ciloxan [Ciprofloxacin 0.3%, Alcon, Fort Worth, TX]) and antimicrobial absorbent dressing changes (TELFA^™^ AMD^™^, blue dots) on post wounding days 5, 6, 8 and 10, and were then harvested on days 5 or 9 (solid black arrows, Biofilm infection). At each harvest time, wounds were analyzed for numbers of bacteria (viable counts and qPCR), biofilm morphology by SEM, re-epithelialization, numbers of infiltrating neutrophils and macrophages, and global gene expression (RNA-Seq transcriptome of wound tissue). The large green triangle indicates the theoretical increase in biofilm for graphical representation only. **(B)** Rabbit ear with six, 6 mm full-thickness dermal excisional wounds.

The experiment schematized in Fig 1 was performed. At each wound harvest time, the endpoints were compared between infected and vehicle control (non-infected) wounds.

### Characterization of the infecting bacteria

#### Viable and qPCR bacterial counts

**Fig 2:**
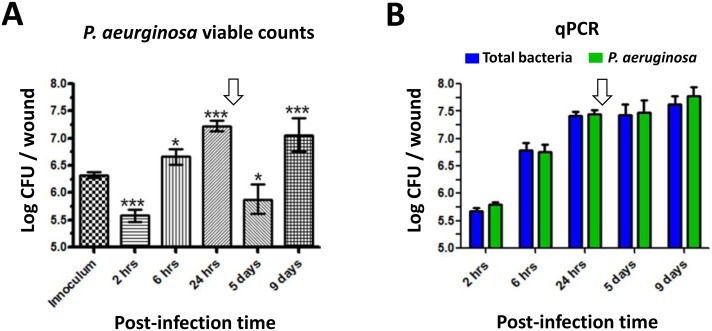
Bacterial counts from wounds. Mean bacterial counts per wound were determined after 10^6^ CFU of *P*. *aeruginosa* PAO1 were inoculated. The vehicle control wounds (PBS-treated) were void of bacteria. **(A)** Viable *P*. *aeruginosa* counts. **(B)** Real-time quantitative polymerase chain reaction (qPCR) of *P*. *aeruginosa* and total bacteria. The arrows indicate the point of Ciloxan treatment. Data represent the total geometric mean and standard error (n> 16). Levels of significance (***, p < 0.001; **, p < 0.01; *, p < 0.05) are shown for the comparison of the mean count at each time point and the mean inoculum count (one-way ANOVA with Newman-Keuls multiple comparison test).

At 2 hrs post-infection, ~10-fold fewer viable counts were recovered than were seeded into the wounds. Between 2 and 6 hrs post-infection, viable and qPCR counts increased ~10-fold and then rose another ~10-fold from 6 to 24 hrs (Fig 2). After topical Ciloxan treatment at 24 hrs post-infection, ~100-fold fewer *P*. *aeruginosa* viable counts were subsequently recovered from the wound on post-infection day 5. Although Ciloxan treatment reduced viable counts, qPCR quantification of genome copy counts were not reduced following Ciloxan treatment, suggesting that the DNA of dead bacteria was incorporated into the wound biofilm, as has been shown previously for *in vitro* biofilms of other species [3, 10]. By post-infection day 9, viable counts rebounded to the peak level observed at 24 hrs post-infection.

#### Biofilm morphology

**Fig 3:**
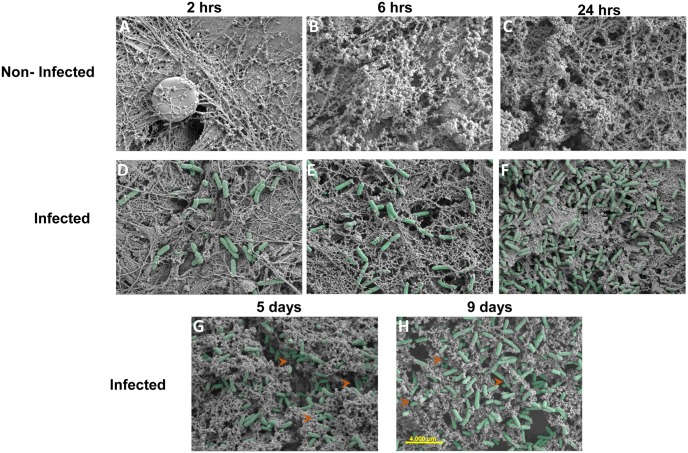
Scanning electron micrographs of *P*. *aeruginosa*-infected and non-infected wounds. The non-infected control wounds at **(A)** 2, **(B)** 6, and **(C)** 24 hrs after receiving PBS are void of bacteria. Bacteria are seen (pseudo-colored) in the infected wounds at **(D)** 2, **(E)** 6, **(F)** and 24 hrs, and **(G)** 5 and **(H)** 9 days post-infection. The red arrows show the area where the bacteria appeared to have penetrated into the tissue. All images were taken at 10K magnification (scale bar is 4 μm).

**Fig 4:**
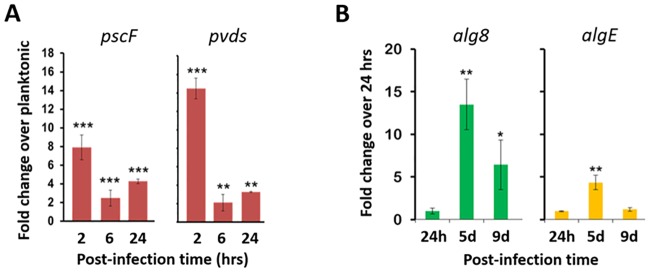
Expression of *P*. *aeruginosa* virulence and biofilm genes. Expression of **(A)** two virulence genes and **(B)** two biofilm synthesis genes of *P*. *aeruginosa* (PAO1) in wounds was determined by RT-qPCR. The virulence gene expression is compared to the level in planktonic cells and the biofilm synthesis gene expression is compared to the level at 24 hrs post-infection (***, p < 0.001; **, p < 0.01; *, p < 0.05; unpaired Student’s t test).

**Fig 5:**
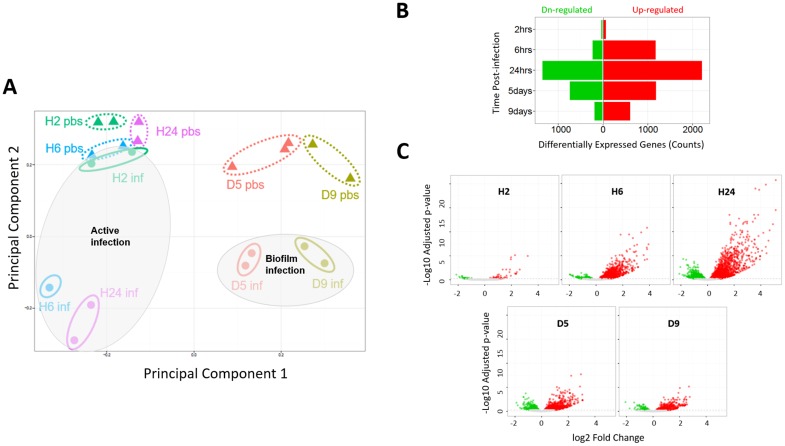
Global gene expression differs between infected and non-infected wounds. **(A)** Principal component analysis of raw transcript counts of non-infected (pbs, triangles surrounded by dotted ellipses) and infected wounds (inf, circles surrounded by solid-lined ellipses) at H2 (green), H6 (blue), and H24 (purple) and D5 (orange) and D9 (army green) post-infection. The acute-active and chronic-biofilm infected wounds are indicated with large grey-shaded ellipses. **(B)** Bar graph and **(C)** volcano plots of genes with significantly changed expression in infected vs. non-infected wounds (green, down-regulated; red, up-regulated; 0.1 FDR).

Similar to the *Pseudomonas* counts (viable and RT-qPCR), *Pseudomonas* cells observed in scanning electron micrographs increased between 2 and 24 hrs post-infection (Fig 3). By days 5 and 9, most bacteria appeared to have penetrated the tissue (as compared to 24 hrs). Unlike *Pseudomonas* biofilms formed in vitro we were unable to see the presence of extracellular matrix among the biofilm cells in wounds. However the biofilm phenotype on day 5 and 9 was confirmed by biofilm-biosynthetic genes expression at their highest levels on day 5 (see below, Fig 4B) and additionally, immune response was suppressed on day 9 as compared to at 24 hrs (see below, Fig 5B and 5C & S1 Fig), characteristic of biofilm infection.

#### *P*. *aeruginosa* virulence and biofilm gene expression

To further characterize the adaptation of the bacteria to the wound and confirm the biofilm phenotype, we quantified the expression of *P*. *aeruginosa* genes (quantitative reverse transcription PCR, RT-qPCR) known to function in virulence and biofilm synthesis. (Fig 4). Two virulence genes—*pscF*, which encodes the type III secretion system needle protein [11–13] and *pvdS*, the iron starvation sigma factor [14–16]—both reached their highest expression level at 2 hrs post-infection: ~8- and ~13-fold, respectively, above the level in planktonic cells. By 6 and 24 hrs post-infection, the expression of both of these virulence genes dropped down to ~3-fold above the planktonic cell level.

In contrast, the two genes involved in biofilm synthesis—*alg8*, a glycosyltransferase in the synthesis of the exopolysaccharide alginate [17], and *algE*, the outer membrane porin through which alginate exits the cell [18]—were most highly expressed on post-infection day 5, being ~14- and ~5-fold elevated, respectively, over their levels at the 24 hr time point. But by day 9 their expression had dropped down to ~5- and < ~2-fold, respectively, of their 24-hr levels. Thus, these data suggest that biofilm was being more actively synthesized on post-infection day 5 and was more mature on day 9.

### Characterization of wound cell infiltration and healing

#### Immune cell infiltration

**Fig 6:**
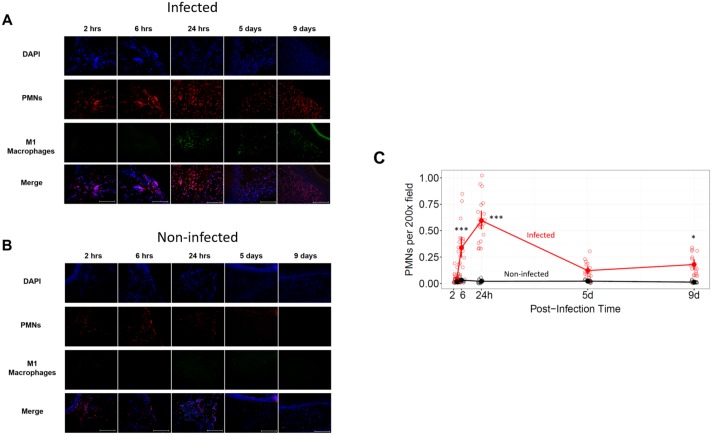
Wound infiltration of neutrophils and M1 macrophages. **(A)** Infected and **(B)** non-infected control wounds were stained for DNA (blue, DAPI), PMNs (red, anti-RPN3/57), or M1 macrophages (cyan-green, anti-HLA DR) Scale bar = 100 μm. **(C)** Quantification of PMNs at the wound edge. Means and 95% confidence intervals are shown. The significance of differences between infected and non-infected wounds are indicated (***, p < 0.001; *, p < 0.05; one-way ANOVA with Tukey’s multiple comparison test; n = 12).

We compared infected with non-infected wounds for PMNs and monocytes/macrophages (M1 and M2) at the wound edge. In non-infected wounds, PMNs were present at all times at similar low levels (Fig 6). However, after infection, the number of PMNs infiltrating the wound significantly increased, firstly at 6 hrs (p < 0.001) and then again over this level at 24 hrs (p < 0.001). In infected wounds treated with Ciloxan, the PMN counts on day 5 did not reach significant levels above those in non-infected control wounds, but they were significantly elevated on day 9 (p < 0.05). In non-infected control wounds, few monocytes and macrophages were seen; however, after infection, monocytes were visible first at 2 hrs post-infection (S2 Fig). Additionally, M1 macrophages were first visible in infected wounds at 24 hrs post-infection, and their abundance was similar on days 5 and 9 (Fig 6). M2 macrophages were not detected in either infected or non-infected wounds (S3 Fig).

#### Re-epithelialization was inhibited by *P*. *aeruginosa* infection

**Fig 7:**
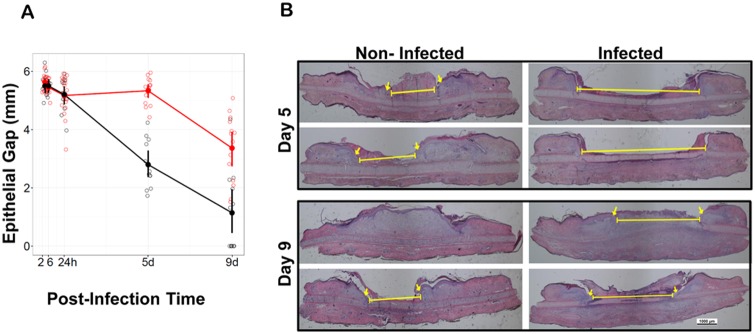
*P*. *aeruginosa* wound infection impairs healing. **(A)** Re-epithelialization as measured by epithelial gap. Means are shown as solid circles with bars representing 95% confidence intervals. The epithelial gap is significantly larger for infected vs. non-infected wounds on day 5 (p = 0.0004) and day 9 (p = 0.004). **(B)** Representative images for the histomorphometry for determining the epithelial gap (n≥9). Yellow line bar define the width of the epithelial gap and the arrows point to the border of a newly formed epithelial tongue. The panel without a yellow line (non-infected, day 9) shows complete closure.

The impact of the *P*. *aeruginosa* infection on healing was evaluated using histology to measure the epithelial gap across wounds (Fig 7). On post-infection day 5, the epithelial gap of non-infected control wounds closed to 3 mm, significantly smaller than the original 6 mm wounds; while the infected-wound gap remained as open as on the day of infection. By day 9, the infected-wound gap was 4 mm, whereas the non-infected wound gap was 1 mm. The infected-wound re-epithelialization was significantly delayed relative to the non-infected control wounds (PBS control) on days 5 (p = 0.0004) and 9 (p = 0.004).

### Transcriptome of the wound edge and proximal tissue

#### Overall differential gene expression between active- and biofilm-infected wounds

Principal component analysis (PCA) was performed on the raw RNA-Seq read count data to evaluate its consistency. The PCA plot shows that the biological replicates are aggregated (Fig 5A, same colored symbols). Additionally, wounds harvested at earlier times (2, 6, and 24 hrs) segregate from wounds harvested at later times (5 and 9 days) across principal component 1. Furthermore, across the second principal component, the non-infected control wounds are segregated from the *P*. *aeruginosa*-infected wounds. Thus, genome-wide gene expression discriminates biological replicates, wound harvest times, and infection status.

Differentially expressed genes (DEGs) between infected and non-infected wounds were identified from the raw RNA-Seq read count data using DESeq2 [19] (S1 File). The fewest DEGs were found at the earliest post-infection time, 2 hrs (Fig 5B & 5C). By 24 hrs post-infection, coincident with the peak level of *P*. *aeruginosa* viable counts, the DEG counts of the wound tissue were greatest, nearly 40-fold more than at 2 hrs post-infection. In wounds infected for 24 hrs and then treated with Ciloxan, the DEGs were subsequently reduced on day 5 to about half the number as before Ciloxan treatment. This drop in DEGs by half coincided with a 10-fold reduction in viable *Pseudomonas* and ~3-fold lower neutrophils than at 24 hrs post-infection. Additionally, the epithelial gap was fully open in infected cells, although it was closed by half in non-infected control wounds; thus, the day-5 DEGs also reflect the delayed healing of infected wounds, whereas at 2, 6 and 24 hrs, the DEGs mostly reflect the acute response to active infection.

From days 5 to 9, the DEGs dropped ~3-fold (Fig 5B) despite *P*. *aeruginosa* viable counts increasing ~10-fold, likely because these viable cells in mature biofilm on day 9 (see Fig 4) were shielded from direct contact with immune and cutaneous cells, resulting in a lower grade inflammatory response, as described below. Additionally, the fewer DEGs on day 9 probably reflect both control and infected wounds being partially re-epithelialized, unlike on day 5 when control wounds had started to close but infected wounds were fully open (see Fig 7).

#### Upregulation of immune-related and downregulation of skin-enriched genes in infected wounds

**Fig 8:**
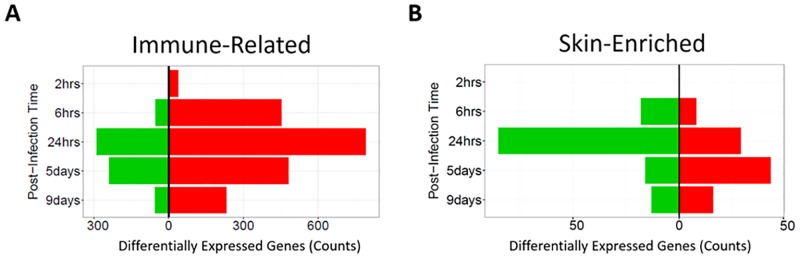
Differentially-expressed immune-related and skin-enriched genes. **(A)** The immune-related genes are from the Comprehensive List of Immune-Related Genes of The Immunology Database and Analysis Portal (ImmPort) ([20]; http://www.immport.org/immport-open/public/home/studySearch?searchTerm=SDY144). **(B)** The skin-enriched genes are comprised of 412 genes with elevated expression in skin compared to other tissue types (www.proteinatlas.org; [21]).

At all post-infection times, the infection induced more up-regulated than down-regulated genes (Fig 5B & 5C), which may be partially due to infiltrating leukocytes (see Fig 6). Considering only genes from the Comprehensive List of Immune-Related Genes of The Immunology Database and Analysis Portal (ImmPort) [20], the ratio of up-regulated to down-regulated genes was even greater (Fig 8A & S2 File). Conversely, skin-enriched genes, which have elevated expression in skin vs. other tissues (www.proteinatlas.org [21]), were mostly suppressed during active infection (6 and 24 hrs). However, they were mostly up-regulated when the inflammatory response had largely subsided on days 5 and 9 (Fig 8B, S3 File). These results suggest that in response to the infection/inflammation that characteristically includes low oxygen and nutrients and oxidative damage, transcription in cutaneous cells is largely suppressed, but is activated when the stress subsides, such as on day 5 when the wounds may have been prepared to close and on day 9 when they had started to close.

#### ncRNA down-regulation in resident cutaneous cells is an early response to active infection, but after biofilm formed and inflammation subsided, they became up-regulated

**Fig 9:**
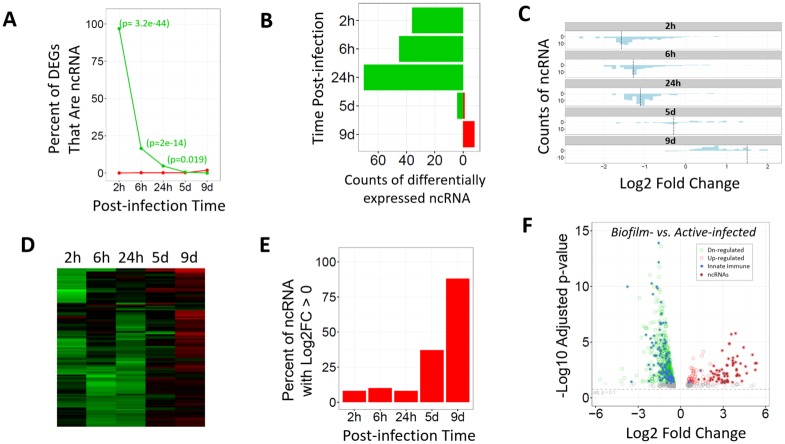
Non-coding RNA levels in infected vs. non-infected wounds. **(A)** Percent of all significantly down-regulated transcripts (green line) and up-regulated transcripts (red line) that are ncRNA (p-value shown in parentheses for Fisher's exact test for overrepresentation of ncRNA). **(B)** Counts of significantly down-regulated (green) and up-regulated (red) ncRNAs in infected wounds (0.1 FDR). **(C)** Histogram of fold changes of 120 ncRNA whose levels changed significantly at least at one post-infection time point (no p-value cutoff). Dotted vertical lines indicate the mean log2FC. **(D)** Heat map of the 120 ncRNAs whose levels changed significantly at least at one post-infection time point. **(E)** Percent of the 120 ncRNAs with Log2 fold change greater than zero (no p-value cutoff). **(F)** Volcano plot of pooled samples from days 5 and 9 compared with the pooled samples from hours 6 and 24, which shows genes all up-regulated (red open circles) and down-regulated genes (green open circles) in the chronic biofilm-infected wounds vs. the acute active-infected wounds. The overlaid solid circles indicate genes of the innate immune response (blue [23]) or ncRNAs (brown, as annotated in the biotype category in Ensembl); FDR = 0.05.

At 2 hrs after infection, there was a total of 37 significantly down-regulated genes, and surprisingly, 36 were ncRNA (annotated in Ensemb, GRCh38.p5l) [22]. Since within our dataset of 14370 annotated genes there are only 525 ncRNA genes, they are highly overrepresented at this earliest time post-infection (Fisher's exact test: p = 3.2e-44, odds ratio = 892). Non-coding RNA genes were also overrepresented among the down-regulated genes at 6 and 24 hrs post-infection, although as the infection intensified over 24 hrs, the proportion of ncRNA genes to protein-coding genes dropped (Fig 9A). Nonetheless, during acute infection at 2, 6, or 24 hrs post-infection, all of the ncRNA that were significantly differentially expressed were down-regulated (Fig 9B). This down-regulation of ncRNAs likely occurred in the resident cutaneous cells, not in infiltrating cells, because as compared to non-infected control wounds, cells that infiltrate infected wounds contribute transcripts.

Subsequently, on days 5 and 9, far fewer ncRNA were significantly differentially expressed, but they became up-regulated (1 of 5 DEGs on day 5, and 9 of 9 DEGs on day 9, were up-regulated; Fig 9B). That the differentially expressed ncRNA were all down-regulated during acute infection (2, 6, and 24 hrs), were partially up-regulated at day 5, and were all up-regulated on day 9 was also seen as a trend for the pool of all 120 ncRNA significantly differentially expressed at least at a single time point (therefore p-values at some time points were non-significant; Fig 9C, 9D and 9E; S4 File). Additionally, pooling samples from days 5 and 9 wounds vs. hours 6 and 24 wounds before calculating differential expression showed that 68% of the 128 significantly up-regulated genes (> 2-fold, p<0.05) in the biofilm-infected wounds on days 5 and 9 were non-coding genes (Fig 9F; S5 File). This is despite ncRNAs comprising only ~4% of all the genes in our dataset.

#### Differential expression of ncRNA types during acute infection

At 2 hrs post-infection, the 36 down-regulated ncRNA of cutaneous cells were largely unique to this time point, with only 4 being differentially expressed at another time point. In contrast, these differed from the subsequently down-regulated ncRNA at 6 and 24 hrs that largely overlapped with each other (see S4 File). The initial ncRNAs down-regulated at 2 hrs were mostly small nucleolar RNAs (snoRNA, both H/ACA and C/D Box classes), miRNA, scaRNA (small Cajal body-specific RNAs), as well as small nuclear RNAs (snRNAs) that were largely RNU6 pseudogenes and whose downregulation preceded the downregulation of skin-enriched genes (see Fig 8B). Subsequently, at 6 and 24 hrs post-infection, coincident with the suppression of skin-enriched genes, a different set of snoRNA and snRNA were down-regulated and the snRNA largely consisted of components of the major and minor spliceosomes.

**Fig 10:**
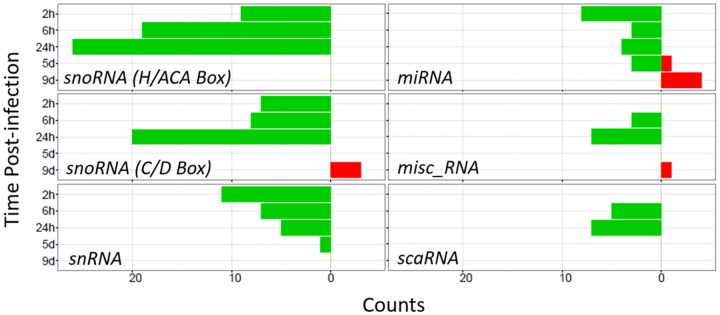
Counts of differentially expressed ncRNAs by type for infected vs. control wounds. Green, down-regulated; red, up-regulated (0.1 FDR). The topmost types of ncRNAs that were differentially expressed are shown.

Among all ncRNA types, snoRNAs were most frequently down-regulated in the actively infected wounds (Fig 10). Most snoRNAs are processed intron fragments and function to guide chemical modifications of other RNAs, mainly ribosomal RNAs, transfer RNAs (tRNAs), and small nuclear RNAs (snRNAs) [24]. The snoRNAs classified as H/ACA Box and C/D Box differ in the chemical modifications they guide. Somewhat more H/ACA Box than C/D Box snoRNAs were down-regulated, but the counts of the members of both classes that were down-regulated increased as the acute infection intensified, coincident with the suppression of skin-enriched gene expression. In contrast, the counts of the down-regulated snRNAs and miRNAs trended toward a reduction as the acute infection intensified from 2 to 24 hrs.

#### ncRNAs of control wounds increased over post-wounding day 3 (from 2 to 24 hrs) and then fell, while ncRNAs of infected wounds did not increase until post-wounding day 8 (post-infection day 5) and then increased further on post-wounding day 12 (post-infection day 9)

**Fig 11:**
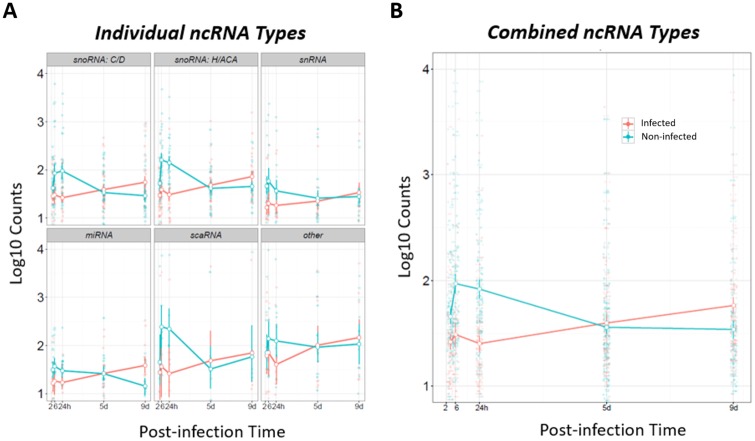
Average raw sequence counts over time for differentially expressed ncRNA genes. **(A)** Plots of the average counts for separate ncRNA types. **(B)** Average counts for all the ncRNA types combined. The means and 95% confidence intervals are plotted. Plotted are the raw sequence counts for the set of 120 ncRNAs that were differentially expressed at least at one time point.

As described above, at post-infection hours 2, 6, and 24, on the third post-wounding day, resident cutaneous cells of infected wounds expressed fewer transcripts of multiple specific ncRNAs of the major ncRNA types relative to non-infected control wounds (Fig 9B, combined types; and Fig 10, types analyzed separately). This differential expression could have come from a post-infection decrease in infected-wound ncRNAs or from a post-infection increase in control-wound ncRNAs. To assess which of these possibilities predominated, we analyzed raw sequence counts of the ncRNAs for infected and control wounds separately. At 2 hrs, the counts of several types of ncRNA were lower in infected wounds than non-infected wounds (Fig 11), consistent with their calculated differential expression. However, between 2 and 24 hrs, the counts of ncRNAs of infected wounds stayed constant, while they increased in control wounds, such that the significant down-regulation of ncRNAs of acutely infected wounds, relative to control wounds, is largely accounted for by control-wound ncRNAs increasing. Following this initial suppression during active infection, the ncRNAs increased from the 24 hrs to the 9 days, while in the non-infected control wounds they decreased, such that by day 9, the infected wounds had significantly higher expression of these ncRNAs that were differentially expressed at one or more time points (Fig 11B). We used RT-PCR to validate these ncRNA levels. Several of the ncRNA could not be validated by RT-PCR because they contained more than one isoform (SNORA1, RNU6-1136P and RNU6-1144P); however, the trends for other ncRNAs were validated (SNORA16 and MIR214; S1 Table).

## Discussion

We hypothesized that insights into the wound response to *Pseudomonas* active and biofilm infections could be gained by analyzing the combined transcriptomes of the multiple cell types of the wound. We found that the genes that were down-regulated in infected compared to sham-infected wounds on post-wound day 3 at 2 hrs post-infection were nearly all ncRNAs. Subsequently, as the active infection intensified over the next 24 hrs, protein-coding genes also became down-regulated, but ncRNAs remained overrepresented among all down-regulated genes (non-coding and protein-coding). Interestingly, the ncRNAs suppressed at 2 hrs were different than those suppressed at 6 and 24 hrs, and at these two later times, the ncRNAs mostly overlapped and included the building blocks of the major and minor spliceosomes.

The differentially-expressed ncRNAs’ raw sequence counts (used to calculate the differential expression) increased in non-infected control wounds, but stayed constant in the acutely infected wounds, indicating that infection suppressed the increase in ncRNAs that occurred in the control wounds that received only PBS on post-wounding day 3. Subsequently, as biofilm formed in the infected wounds, the ncRNAs increased (on days 5 and 9), but decreased in control wounds, such that on day 9, the ncRNA counts of infected wounds, which were half open, exceeded those of the control wounds, which were nearly closed.

We were surprised that ncRNAs increased in wounds on post-wounding day 3 between 2 and 6 hours after they received only PBS (S6 File). This phenomenon may have resulted from the PBS providing moisture and pH buffer that stimulated healing [25–27]. Consistent with this hypothesis, protein-coding genes expressed more at 6 hrs than at 2 hrs in these PBS-treated control wounds were overrepresented among gene sets associated with activated 'formation of skin' as well as 'differentiation of skin' (Ingenuity Pathway Analysis, p-value 1.31E-10 and p-value 1.43E-05, respectively); and, their predicted top activated upstream regulator was the epidermal growth factor receptor (EGFR). Thus, between 2 and 6 hrs after receiving PBS on day 3, the increase in transcript counts of multiple classes of ncRNA may coincide with a shift from the inflammation phase to the proliferation phase of healing.

The gene expression program undoubtedly shifted in cells of control wounds as they closed between 2 hrs and 5 days post-PBS treatment. During this time their ncRNA counts first rose from 2 to 24 hrs, then they fell by day 5, when the wounds were already half closed. In contrast, infected wounds apparently failed to fully transition to the proliferation phase, as they were still fully open on day 5. And from 2 to 24 hrs post-infection, their ncRNA transcripts did not change, but then rose on day 5, and then again on 9. This rise in ncRNA transcripts as the infected wounds attempted to close may hypothetically be due to keratinocytes at the wound edge continuously trying to proliferate and migrate in the face of biofilm, similar to the situation in chronic diabetic ulcers wherein keratinocytes are highly proliferative but lack markers of migration and epidermal differentiation [28]. Thus, as the wounds became chronically infected with biofilm, the coincident increase in ncRNA transcripts might reflect wound cells repeatedly exiting the metabolically-suppressed inflammation state to proliferate, mostly futilely.

The down-regulated C/D Box snoRNA transcripts at 2 hrs post-infection had a size distribution like all annotated C/D Box snoRNA genes, while at 6 and 24 hrs, there was a shift in the distribution to include longer less abundant C/D Box snoRNAs (S4 Fig). A similar temporal shift in sizes was observed for snRNA: at 2 hrs, the differentially-expressed snRNAs were mostly RNU6 pseudogenes, an abundant class of snRNA with unknown function; while at 6 and 24 hrs, the down-regulated snRNA were larger and consisted mostly of the snRNA building blocks of the major and minor spliceosomes. These snRNAs have been previously shown to be down-regulated in response to intracellular infection with *Shigella*, which affected spliceosomal snRNA maturation, dependent on the activation of a metabolic stress pathway [29]. Our studies suggest the possibility of a similar effect in response to *Pseudomonas* infection, and the altered expression of the major and minor spliceosome components could possibly influence alternative splicing, as previously suggested [30].

In infected wounds on post-wounding day 3, the suppressed ncRNA transcripts could potentially be a byproduct of the stress-induced inhibition of translation, or could alternatively function as a checkpoint in the regulation of the stress-response [29]. The integrated-stress-response pathway starts with a general shutdown and reprogramming of protein synthesis and involves reprogrammed transcription for overcoming stress [6, 23, 31]. In our study, early at 2 hrs after *Pseudomonas* infection, a lowering of ncRNAs preceded the up-regulation of the stress-activated transcription factors, ATF3 and ATF6, and the down-regulation of skin-enriched genes at 6 and 24 hrs.

Non-coding RNAs have been shown to play a role in stress-responses [32–35]. For example, the stress sensor PKR (protein kinase RNA-activated), long known to be activated by viral dsRNA, was activated by snoRNA in response to metabolic stress [36]. Additionally, miRNAs function in stress responses [37], being enriched along with their mRNA targets, Argonaute, and miRNA-protein complex components in stress granules, which form in response to various environmental stresses and translation inhibition [23]. The formation of stress granules can be triggered by the phosphorylation of the translation initiation factor eIF2α, which is governed by five stress-sensing kinases, one being the stress sensor PKR [37]. Stress granules are often detected docked to P-bodies—cytoplasmic structures containing small mRNAs, proteins, and ncRNAs associated with post-transcriptional regulation [38], which have been implicated in the regulation of RNA metabolism during viral and bacterial infections [39]. The ncRNA patterns we have identified could reflect such processes and provide clues to transcriptional regulatory pathways underlying responses to stress and changes in cell states.

There are many limitations and uncertainties in interpreting transcriptomic data of a tissue’s heterogeneous cell populations in mixture. In our experiments, we interpret the down-regulated genes during acute infection (2 to 24 hrs post-infection) to have been down-regulated in the resident cutaneous cells, rather than in the infiltrating cells, because the infiltrating cells contribute transcripts to the infected wounds as compared to control wounds. An alternative interpretation is that infiltrating cells dilute the RNA of the wound tissue, making its gene expression appear to be generally down-regulated. Such an effect appears not to explain our results because at 2 hrs post-infection, gene expression was not down-regulated in general, rather, ncRNAs were preferentially down-regulated, and they remained over-represented among down-regulated genes at 6 and 24 hrs post-infection. Also, the set of ncRNAs at 2 hrs post-infection switched to a different set at 6 and 24 hrs post-infection which largely overlapped. Additionally, the counts of some down-regulated ncRNA classes decreased (snRNA and miRNA) between 2 and 24 hrs although the number of infiltrating cells increased. Furthermore, between 6 and 24 hrs post-infection, the skin-enriched protein-coding genes were down-regulated ~4.5 fold, while infiltrating PMNs increased only ~2-fold.

### Conclusions and hypotheses

Our data show that at 3 days post-wounding, the genes down-regulated soonest after infection, at 2 hrs post-infection, were nearly exclusively ncRNAs, and they were down-regulated prior to the general down-regulation of skin-enriched coding gene expression at 6 and 24 hrs post-infection. This result suggests the hypothesis that the ncRNAs that are down-regulated soon after infection are part of a mechanism that regulates the response of resident cutaneous cells to infection. Additionally, in non-infected wounds, from 2 to 24 hrs after the day 3 wounds received the PBS vehicle alone (i.e., sham-infection), the raw counts of the differentially expressed ncRNA transcripts increased, but they did not increase in the wounds with biofilm infection until later when they were starting to close. This result suggests the hypothesis that these ncRNAs may be part of a mechanism for regulating changes between cell states, such that from the metabolically suppressed state of inflammation to the proliferation phase of wound healing.

## Methods

### Animal protocol

Adult female New Zealand white rabbits (3-5kg) from Charles River Laboratories International, Inc. were acclimated to standard housing, fed ad libitum, and kept in individual cages under constant temperature (22°C) and humidity with a 12-hour light dark cycle. A total of 89 animals were used for this study. Two biological repeats were used for each time point, except for the 5-day post-infection time point that used 3 biological repeats. A preemptive dose of analgesic Buprenorphine HCL SR (Zoopharm; 0.3–0.5 mg/kg) was administered subcutaneously 15 minutes earlier to the anesthetic induction. Thereafter, it was administered as needed, based on the clinical assessment of pain and/or distress. Prior to the surgery for creating full-thickness dermal wounds, rabbits were anesthetized by intramuscular injection of a mixture of ketamine (22.5mg/kg) and xylazine (3.5mg/kg). Ears were shaved, sterilized with a betadine scrub and 70% ethanol and injected intradermally with 1% lidocaine with epinephrine before making six 6-mm-diameter full-thickness dermal wounds down to the perichondrium on the ventral surface, which were then dressed with Tegaderm [40, 41]. After wounding, the animals were randomized into two groups: vehicle control (non-infected) and infected. Each biological repeat at 2, 6, and 24 hrs post-sham-infection (control groups) consisted of 3 animals, while for the infected groups they consisted of 5 animals. At days 5 and 9, the biological repeats consisted of 3 animals for non-infected, while the infected groups consisted of 4 animals. The number of animals used for each biological repeat for each time point is shown in S2 Table. On post-wounding day (PWD) 3, the Tegaderm was removed, and wounds of the infected group received 10^6^ *P*. *aeruginosa* CFU in 10 μl PBS, while non-infected control wounds received just PBS. Wounds of both groups were subsequently re-dressed with Tegaderm. On PWD 4, wounds were treated with topical Ciloxan to select for biofilm (in the infected wounds). On PWD 5, the Tegaderm was removed and replaced with TELFA^™^AMD^™^, an antimicrobial non-adherent dressing, which was changed on post-wounding days 6, 8 and 10 [6]. For studying active infection, wounds were harvested at post-infection times 2, 6, and 24 hours. For studying biofilm infection, the Ciloxan-treated wounds were harvested on post-infection days 5 and 9. At each wound harvest time, animals were euthanized by intravenous injection of a euthanasia solution (Fatal-Plus TM). All animals were monitored minimum of twice daily for general appearance, behavior, and signs of wound infection. Body weight was obtained daily as part of their assessment of pain and/or distress. All dressings were checked every day throughout the entire protocol.

### *Pseudomonas* strain and growth conditions

*P*. *aeruginosa* (PAO1, University of Washington subline) for the inoculum was grown on blood agar plates overnight at 37°C before being subcultured into 10 mL of tryptic soy broth (TSB) and grown at 37°C until log-phase. They were then washed with PBS, centrifuged (4,000 rpm for 10 minutes at 20°C), and resuspended in PBS to an OD_600_ equal to 10^6^ CFU/10 μl.

### Total RNA extraction

The dorsal skin of the ear was removed prior to harvesting the wound using an 8-mm punch. The harvested tissue was immediately frozen in liquid nitrogen. To obtain total RNA, the frozen tissues were homogenized in TRIzol (Life Technologies) using the T25 ULTRATURRAX at 20,000rpm (IKA, Germany). The RNA was purified using an RNEasy Mini-Kit (Qiagen, MD). Genomic DNA was removed by treatment with DNAse I (Ambion), and the absence of DNA contamination was confirmed using a control without reverse transcriptase and demonstrating a Ct value 10 cycles higher than the reverse transcribed samples. The RNA was quantified (A260/A280, NanoDrop, Thermo Scientific, Waltham, MA) and checked for purity (2200 TapeStation, Agilent Technologies, Cedar Creek, TX). The purified RNA was reverse transcribed to cDNA using the iScript Select cDNA synthesis kit (Bio-Rad). The RNA was isolated from seven of the 12, 8-mm biopsy punches of the wounds (to include the wound edges) harvested from each animal. The remaining five were used for viable counts, SEM, epithelial gap and immunohistochemistry. The RNA from each wound was individually isolated and the purity was checked before pooling. Each RNA sequence determination was from RNA pooled from 28–30 infected wounds or 18–21 non-infected wounds.

### Quantitative RT-PCR

Quantitative real-time PCR was performed using the SYBR green master mix (Bio-Rad) with specified primers (S3 Table) and was analyzed using the StepOne System (Applied Biosystems). Primer sets were provided by Applied Biosystems (Inventoried Assays-on-Demand^™^). Expression of mRNA in wound tissue was determined by the 2^-ΔΔCt^ method, with normalization by beta-actin as the housekeeping gene for wounds, and by 16S and FabD as the housekeeping genes for *P*. *aeruginosa*. The TaqMan^™^ probe-based gene expression analysis used primers and TaqMan specific probes for: IL-1β (assay number: Oc03823250_s1), IL-6 (assay number: Oc04097051_m1), TNFα (assay number: Oc03396940_m1), and β-actin (assay number: Oc03824857_ml) and primers for ncRNAs were listed in S3 Table. These RT-qPCR results validated the RNA-Seq data for coding (S1 Fig) and ncRNA genes (S1 Table).

### Bacterial counts from wounds

Harvested wound tissues were placed in tubes pre-filled with homogenizer beads (Roche, Indianapolis, IN). After adding 1 mL of PBS, the tubes were homogenized for 90 s at 5000 rpm in a MagnaLYSER homogenizer (Roche, Indianapolis, IN). The homogenate was removed for determination viable counts (500 μl) and for isolating bacterial genomic DNA for determinations of *Pseudomonas* and total bacterial counts by PCR (200 μl). The viable counts were determined as previously described [3], and control wounds were similarly assayed to ensure their sterility.

For the determination of total cell counts by PCR, bacterial DNA was isolated from 200 μl of the homogenate (cleared by centrifugation, 3 min at 4°C at 14,000 rpm in an Eppendorf 5417R), using the DNeasy Blood & Tissue Kit (Qiagen, Valencia, CA), according to the manufacturer's instruction. Total *P*. *aeruginosa* cell counts were quantified by PCR analysis of the outer membrane lipoprotein gene *oprL* [42]. Total bacterial load was quantified by PCR analysis of 16S rDNA [43]. The primers and probes were synthesized by Applied Biosystems (Carlsbad, CA) and were listed in S3 Table. For these determinations, the standard curve was generated using the genomic DNA that was isolated from mid-log growth phase *P*. *aeruginosa* cultures (quantified using the Quant-iT ds DNA BR Assay Kit, Invitrogen).

### Scanning electron microscopy

Wound tissues were fixed with 2.5% phosphate-buffered glutaraldehyde for at least 12 hours at 4°C. They were dehydrated in a graded series of cold ethanol/water mixtures, increasing from 10%, 20%, 30%, 50%, 70%, 80%, 90%, 95%, to 100% ethanol, 10 min. each before being dehydrated by critical point drying (EMS 850, Electron Microscopy Science Hatfield, PA). The samples were then coated with a gold/palladium target (Hummer 6.2 Sputter Coater, Anatech USA, Hayward, CA) and observed with a field emission scanning electron microscope operated in high vacuum mode at 2 kV (SIGMA VP40, Carl Zeiss, Inc., Germany).

### Histology and immunohistochemistry

Wounds excised with a 10-mm punch were bisected at their largest diameter, fixed in formalin, embedded in paraffin, cut into 4.5-μm sections, and stained with hematoxylin and eosin (H&E) for subsequent light microscopy (Nikon, Eclipse 55i and the attached Nis-Element BR 4.13.00, Japan). The histomorphometric parameter, epithelial gap (EG), for evaluating wound healing, was determined as previously described [41]. For immunohistochemistry, the formalin-fixed paraffin-embedded sections were deparaffinized with xylene and rehydrated through 100, 95, and 70% ethanol followed by incubation in deionized water for 5 minutes. They were then incubated in 100 mM glycine for 10 min before being blocked for 1 hr with 5% fetal bovine serum (FBS) in Tris-buffered saline. PMNs staining was done by using a mouse anti-rabbit RPN3/57 antibody (sc-59376; Santa Cruz Biotechnology, CA) that was directly conjugated with a fluorochrome (Readilink antibody labeling kit Bio Rad, CA) to minimize non-specific staining. The sections were incubated in the conjugated antibody (1:100) for 2 hours at room temperature. To stain monocytes and macrophages, we used the mouse anti-macrophage antibody MAC387 (ab22506; Abcam) (1:20) vs. the control, mouse IgG (H+L) (Life Technologies), for 1 hour at room temperature, at a final concentration of (1:500). To stain M1 and M2 Macrophages, Anti-HLA DR (for M1; LN-3, Abcam) and anti-human CD206 (for M2, MCA2155, AbD Serotec) mouse antibodies were used, which were directly labeled using Qdot Secondary Antibody Conjugates (Life Technologies, CA) [44, 45]. Incubations of sections with anti-HLA DR (1:50) and anti-CD206 (1:50) were for 2 hours at room temperature. Qdot-labeled mouse IgG1 isotype control (Abcam) was used as the control. The slides were counterstained/mounted with Prolong Gold antifade regent that contained the fluorescent stain 4’,6-diamidino-2- phenylindole (DAPI; Life Technologies) and were photographed under 400X magnification using a fluorescence microscope (Axioplan 2, Zeiss, Germany) [46].

### RNA sequencing

Total RNA was submitted to Otogenetics Corporation (Norcross, GA USA) for RNA-Sequencing. Briefly, the integrity and purity of total RNA were assessed by OD260/280 using the Agilent Bioanalyzer. Total RNA (5 μg) was depleted of rRNA using the RiboZero Magnetic Gold Kit Epidemiology kit (Epicentre). The resulting RNA was converted to cDNA using the TruSeq Stranded Total RNA Sample Preparation kit (Illumina). The resulting cDNA was purified, fragmented by sonication (BioRuptor, Diagenode, Inc.), and profiled using the Agilent Bioanalyzer. Illumina libraries were made from the qualified, fragmented cDNA using the SPRIworks HT Reagent Kit (Beckman Coulter, Inc.) on the Biomek FXp. The quality, quantity, and size distribution of the Illumina libraries were determined using an Agilent Bioanalyzer 2100 or Tapestation. The libraries were then submitted for Illumina HiSeq2500 sequencing. Paired-end 106 or 126 nucleotide reads were generated and checked for data quality using FASTQC (Babraham Institute, Cambridge, UK).

### Bioinformatic analysis of RNA-Seq reads

Illumina generated RNA-Seq reads were mapped to the rabbit genome using the software STAR—an ultrafast universal RNA-Seq aligner (version STAR_2.4.2a) [47]. Top-level unmasked DNA sequences of the rabbit genomic sequences were downloaded from the Ensembl public database [48]. The gene annotation file required by the STAR to generate read counts per gene was downloaded from the same site [49]. A summary of the STAR mapping results for all the samples is included in the S7 File. Strand-specific read count outputs from the STAR mapping were then subject to pair-wise differential gene expression analysis using DESeq2 Bioconductor package version 1.10.1 [19] under the R environment version 3.2.2 [50]. The human orthologs of rabbit genes were identified using the Ensembl database (http://useast.ensembl.org/info/genome/compara/homology_method.html). The comparison of active- and biofilm-infected wounds (pooled samples: 6 and 24 hrs vs. 5 and 9 days) for differential expression was done using EdgeR [51] in NetworkAnalyst [52]. The differentially expressed genes were analyzed for enrichments in genes in pathways or biological functions using Ingenuity Pathway Analysis (Qiagen) in which the *p* values indicate the significance of overlaps between gene sets using Fisher's exact test.

### Statistical analysis

Statistical analysis was performed using GraphPad Prism software and the R software environment for statistical computing and graphics [50]. False discovery rate of 0.1 was used throughout the study unless otherwise indicated.

### Ethics Statement

The animal protocol number A-14-005 was approved by the Institutional Animal Care and Use Committee (IACUC) at the US Army Institute of Surgical Research (USAISR, Fort Sam Houston, TX) on 19 November 2013. All animals received care in strict compliance with the 2011 *Guide for the Care and Use of Laboratory animals* by the National Research Council and were maintained in an Association for Assessment and Accreditation of Laboratory Animal Care International (AAALAC, Int.)- accredited facility at the USAISR.

## DOD Disclaimer

KPL is an employee of the U.S. Government. The work presented is part of his official duties. Title 17 U.S.C. §105 provides that ‘Copyright protection under this title is not available for any work of the United States Government.’ Title 17 U.S.C. §101 defined U.S. Government work as work by a military service member or employee of the U.S. Government as part of that person’s official duties. The opinions or assertions contained herein are the private views of these authors and are not to be construed as official or as reflecting the views of the Department of the Army or the Department of Defense.

## Supporting Information

S1 Fig RT-qPCR validation of the expression of coding genes determined by RNA-Seq. Inflammatory cytokine mRNA levels in wound tissue were measured at 2h, 6h, 24h, 5d and 9d after wounds were infected with *P*. *aeruginosa* (PAO1) or sham-infected, as measured by **(A)** RT-PCR and **(B)** RNA-Seq. The significance of the RT-PCR mRNA levels of infected wounds compared to the control wounds: * *p*<0.05, **p<0.01, ***p<0.001; unpaired Student’s t test; n = 15–30 wounds/group.
(TIF)

S2 Fig Wound infiltration of monocytes and macrophages. **(A)** Infected and **(B)** non-infected wounds were stained for the nucleus (blue, DAPI), Monocytes and macrophages (cyan-green, MAC387) scale bar = 100 μM.
(TIF)

S3 Fig Wound infiltration of M2 macrophages. **(A)** Infected and **(B)** non-infected wounds were stained for the M2 macrophages (orange, CD206).
(TIF)

S4 Fig Density of sizes of differentially expressed ncRNA genes compared to all annotated ncRNAs. All available annotated ncRNAs of the human genome (from Ensembl) are indicated by the red bars and the differentially expressed ncRNAs in our experiment are indicated by the blue bars. The gene names with underlining overlap between 6 and 24 hrs are depicted in each figure. Length of the genes is shown in nucleotides size.
(TIF)

S1 File Differentially expressed genes between infected and non-infected wounds. (XLSX)

S2 File Differentially expressed genes between infected and non-infected wounds filtered for immune-related genes. (XLSX)

S3 File Differentially expressed genes between infected and non-infected wounds filtered for skin-enriched genes. (XLSX)

S4 File Differentially expressed ncRNA genes between infected and non-infected wounds. Only significant values are shown.
(XLSX)

S5 File Differentially expressed genes between infected and non-infected wounds for pooled samples (hrs6&24_vs_days5&9). (XLSX)

S6 File Differentially expressed genes between hour 6 and hour 2 after receiving PBS. (XLSX)

S7 File Read counts per gene for all samples. (XLSX)

S1 Table RT-qPCR Validation of RNA-Seq Expression Data for ncRNAs. (XLSX)

S2 Table Study design for transcriptome profiling of rabbit ear wound responses to *P*. *aeruginosa* infection. (PDF)

S3 Table List of primers and probes used in this study. (PDF)
