# Supplementary figures and images for: RNA-Seq Transcriptomic Responses of Full-Thickness Dermal Excision Wounds to Pseudomonas aeruginosa Acute and Biofilm Infection
Source: PLoS One. 2016 Oct 28;11(10):e0165312. doi: 10.1371/journal.pone.0165312 (PMC5085052; doi:10.1371/journal.pone.0165312)

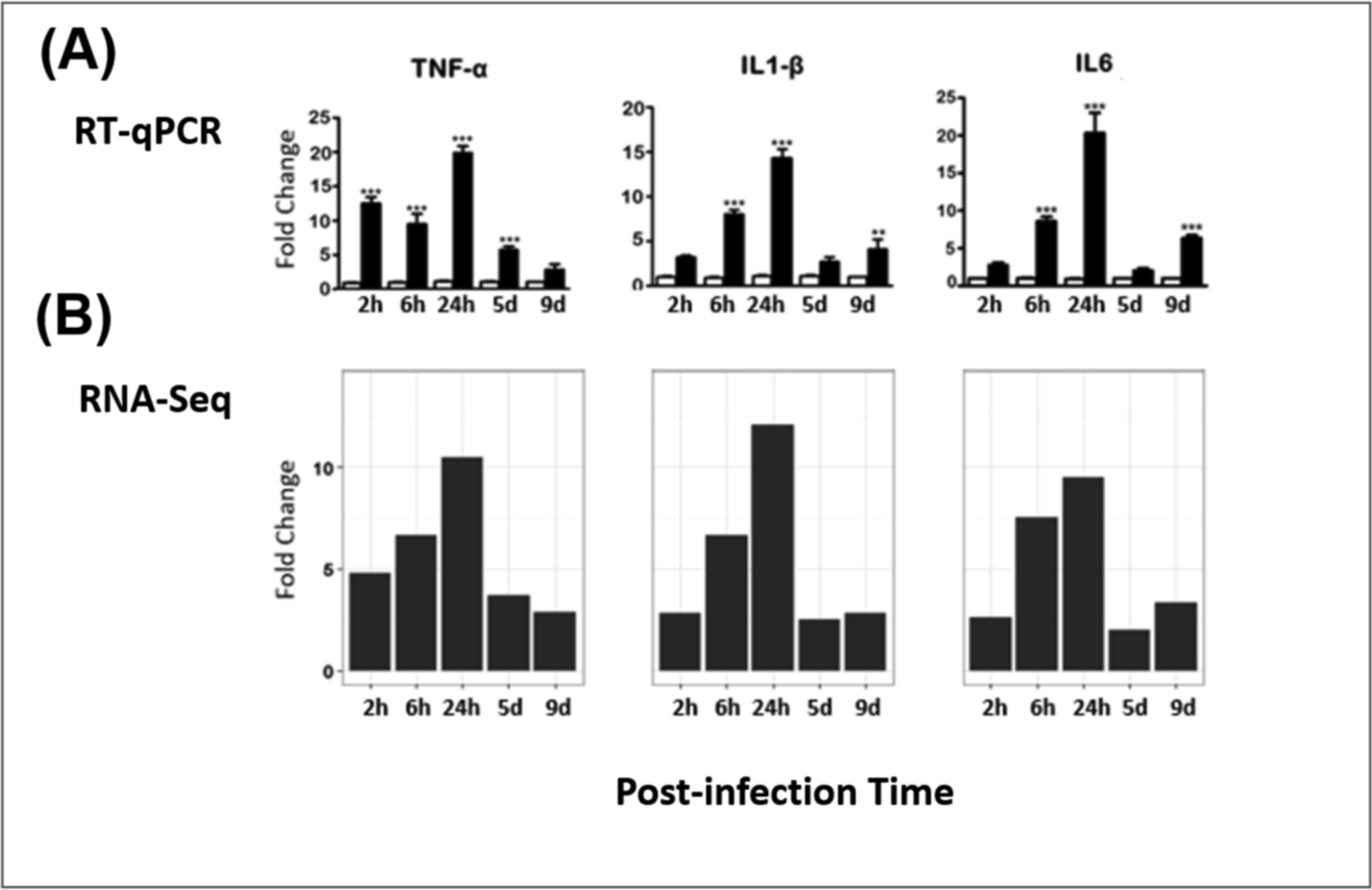

Supplement: S1 Fig — Inflammatory cytokine mRNA levels in wound tissue were measured at 2h, 6h, 24h, 5d and 9d after wounds were infected with P. aeruginosa (PAO1) or sham-infected, as measured by (A) RT-PCR and (B) RNA-Seq. The significance of the RT-PCR mRNA levels of infected wounds compared to the control wounds: * p<0.05, **p<0.01, ***p<0.001; unpaired Student’s t test; n = 15–30 wounds/group. (TIF) [file pone.0165312.s001.tif]

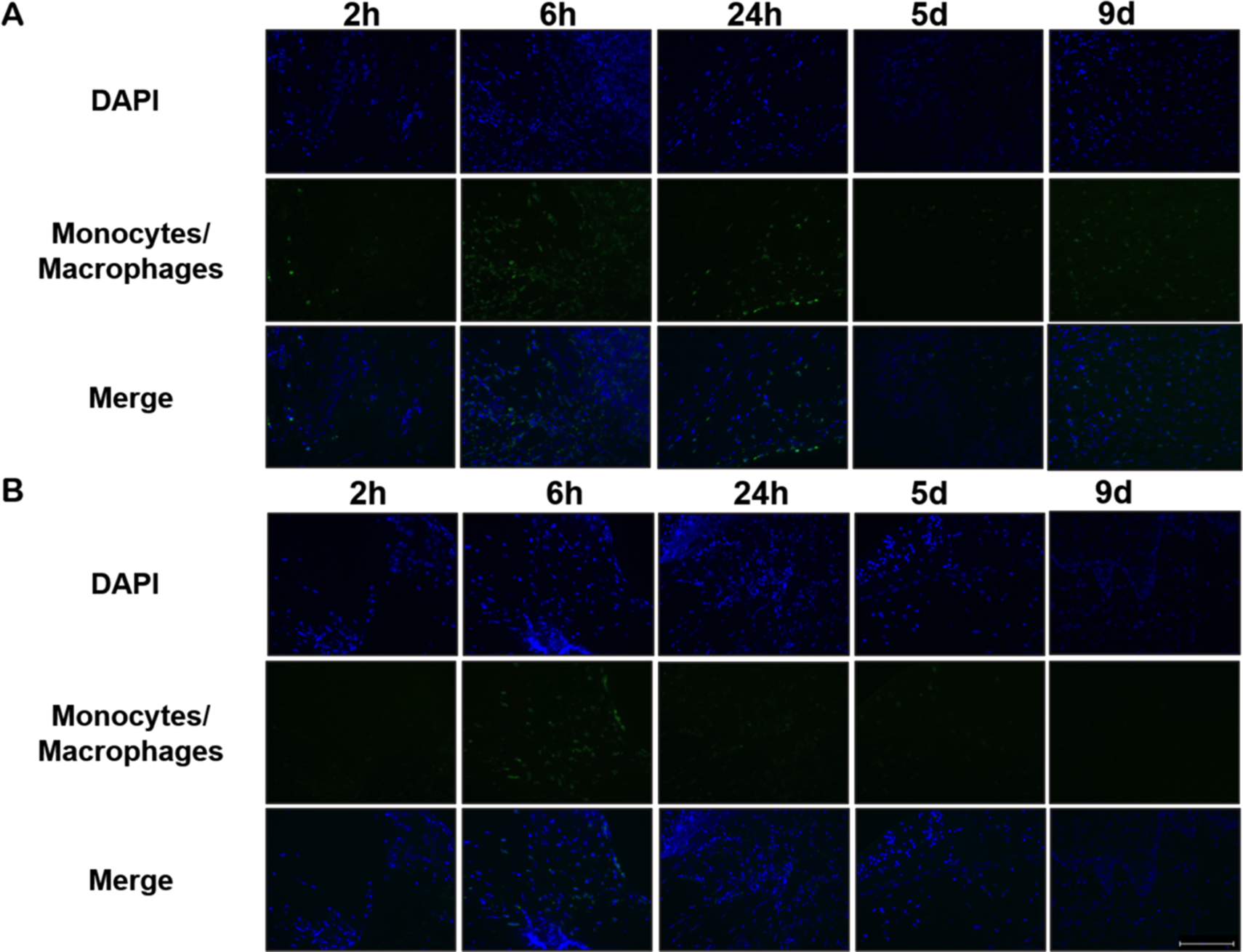

Supplement: S2 Fig — (A) Infected and (B) non-infected wounds were stained for the nucleus (blue, DAPI), Monocytes and macrophages (cyan-green, MAC387) scale bar = 100 μM. (TIF) [file pone.0165312.s002.tif]

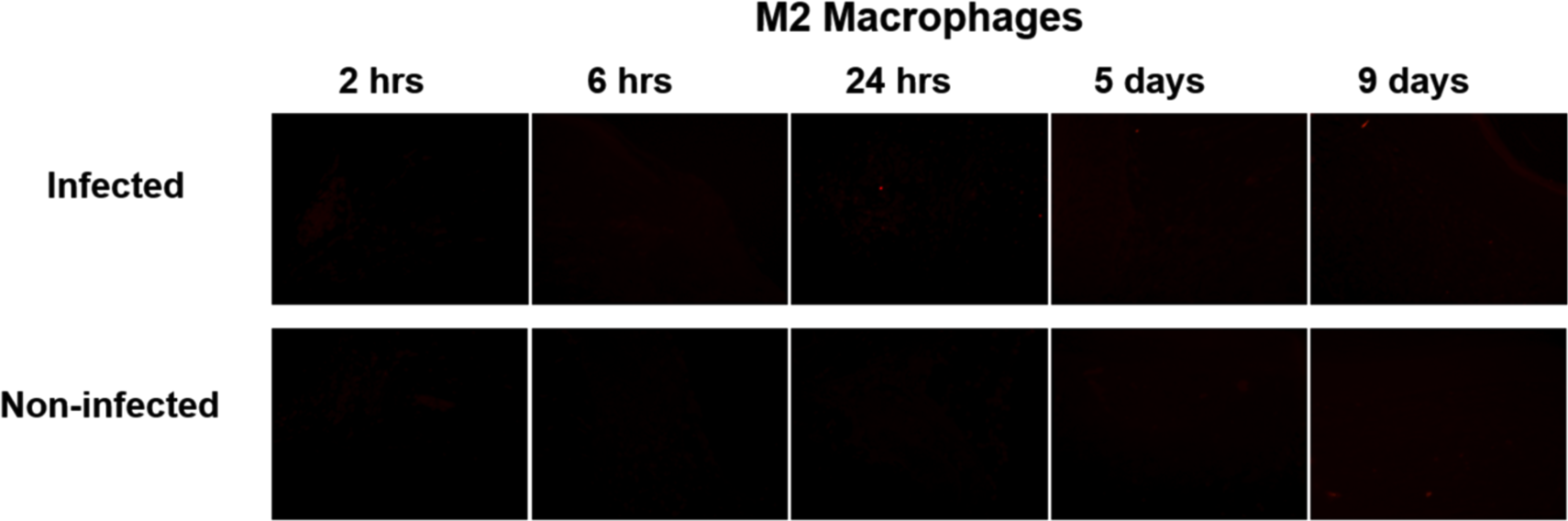

Supplement: S3 Fig — (A) Infected and (B) non-infected wounds were stained for the M2 macrophages (orange, CD206). (TIF) [file pone.0165312.s003.tif]

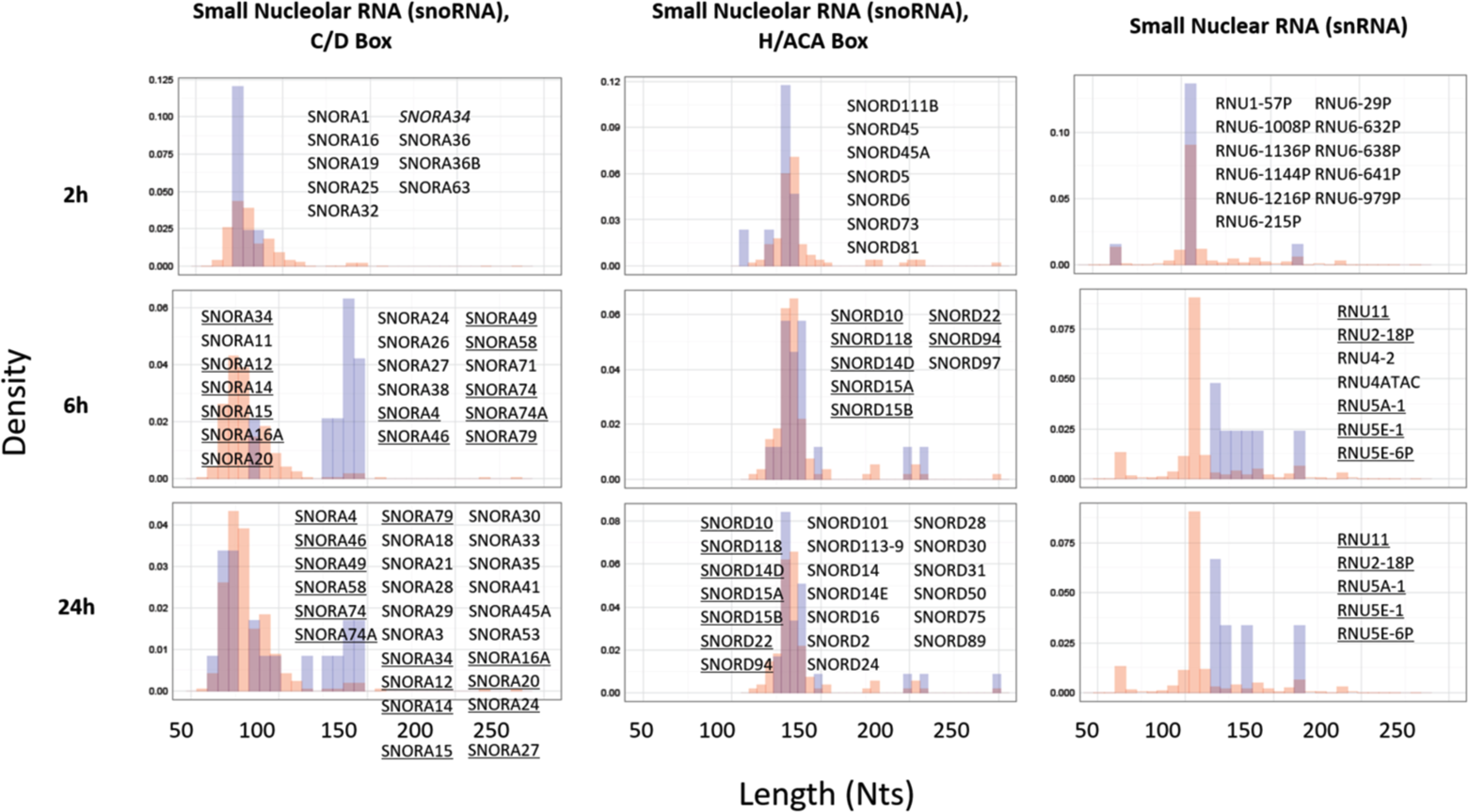

Supplement: S4 Fig — All available annotated ncRNAs of the human genome (from Ensembl) are indicated by the red bars and the differentially expressed ncRNAs in our experiment are indicated by the blue bars. The gene names with underlining overlap between 6 and 24 hrs are depicted in each figure. Length of the genes is shown in nucleotides size. (TIF) [file pone.0165312.s004.tif]
