# Supplementary material for: RNA-Seq Transcriptomic Responses of Full-Thickness Dermal Excision Wounds to Pseudomonas aeruginosa Acute and Biofilm Infection
Source: PLoS One. 2016 Oct 28;11(10):e0165312. doi: 10.1371/journal.pone.0165312 (PMC5085052; doi:10.1371/journal.pone.0165312)
Supplement: S2 Table — (PDF) [file pone.0165312.s013.pdf]

S2_Table: Study design for transcriptome profiling of rabbit ear wound responses to *P .aeruginosa* infection.

Group	Biological Repeats	CFU Inoculated per Wound	Number of Rabbits[a] used in the study						Sub-totals
			[b]PWD0	PWD3 (2 h post-infection)	PWD3 (6h post-infection)	PWD4 (24 h post-infection)	PWD8 (5 d post-infection)	PWD12 (9 d post-infection)	
**1**	A	0	3	3	3	3	3	3	18
**2**		1×10^6	0	5	5	5	4	4	23
**3**	B	0	3	3	3	3	3	3	18
**4**		1×10^6	0	5	5	5	4	4	23
**5**	C	0	-	-	-	-	3	-	3
**6**		1×10^6	-	-	-	-	4	-	4
	Total Rabbits used in this study								89

[a] 12 (6 mm) full thickness dermal excisional wounds were produced per rabbit (6 wounds per ear). All wounds in control and experimental animals were treated the same.
[b]PWD is Post Wounding Day
