# Supplementary material for: RNA-Seq Transcriptomic Responses of Full-Thickness Dermal Excision Wounds to Pseudomonas aeruginosa Acute and Biofilm Infection
Source: PLoS One. 2016 Oct 28;11(10):e0165312. doi: 10.1371/journal.pone.0165312 (PMC5085052; doi:10.1371/journal.pone.0165312)
Supplement: S3 Table — (PDF) [file pone.0165312.s014.pdf]

S3_Table. List of primers and probes used in this study

Name	Seq 5' to 3'
*fabD*F	GGTCCAGAATGGTCCTGAAGAG
*fabD*R	CGATCGAAACCGTAAGGATGGC
16SF	CAAAACTACTGAGCTAGAGTACG
16SR	TAACATCTCAAGGATCCCAACGGCT
pscF-F	GCAGATATTCAACCCCAACC
pscF-R	GATGACCGACCACTTGTTGA
pvdS-F	GATAACCGTACGATCCTGGTGAAG
pvdS-R	GACGATCTGGAACAGGTAGCTGAG
alg8-F	CAAGGATTTCATCCTGCTTATCGG
alg8-R	GAAACTGGTGACCATCAGGAACA
algE-F	GACAACCTCGACAAGACCTATACC
algE-R	CTCGAACGATAGTTGTAGGCATCG
oprL-F	ATGGAAATGCTGAAATTCGGC
oprL-R	CTTCTTCAGCTCGACGCGACG
oprL-Probe	(6FAM)-5'-TGCGATCACCACCTTCTACTTCGAGT-3'-MGBNFQ
16S rDNA-F	TCCTACGGGAGGCAGCAGT
16S rDNA-R	GGACTACCAGGGTATCTAATCCTGTT
16S rDNA-Probe	(6FAM)-5'-CGTATTACCGCGGCTGCTGGCAC-3'-MGBNFQ.
SNORA16-F	TCTGTTGTCCCAGGTAAGCA
SNORA16-R	CAGAAGAGTAGCCACAAAGGGA
MIR214-F	GCTGGACAGAGTTGTCATGTG
MIR214-R	TGCTGTACAGGTGAGCGGAT
SNORA1-F	GCACTGTTAAACATGGTGTCCA
SNORA1-R	TGCTGTGATGGAAGCATAACCT
RNU6-1136P-F	TCACTTTGGCAGCACATATACTAA
RNU6-1136P-R	TTTGCATGTCATCCTTGTGC
RNU6-1144 P-F	TGATGTTGGCAGCACATATACTA
RNU6-1144 P-R	GAATTTGTGTGTCATCCTTGCAC
